# Guest Editor's Concluding Remarks—Advances in Usage of ANN, Discussion of an Unsolved Problem, and Some Differences between Papers Written by Engineers and by Physicians

**DOI:** 10.3390/s91008126

**Published:** 2009-10-16

**Authors:** Michael W. Retsky

**Affiliations:** Harvard Medical School, Children's Hospital, Karp Family Research Labs 12, 300 Longwood Ave., Boston, MA 02115, USA; E-Mail: michael.retsky@gmail.com

I take this opportunity to discuss a few things that I have learned from being Guest Editor of this special issue of Sensors devoted to Neural Networks and Sensors. The advancement in artificial neural network (ANN) technology is very impressive. The wide variety of fields in which this technology applies in the form of practical applications to clearly identifiable real problems demonstrates that ANNs are being routinely used to solve non-trivial problems. I mention that because A. K. Dewdney wrote in 1997 that while ANNs have been used to solve a few toy problems, he was surprised that anyone takes them seriously as general problem-solving tools [1]. The ANN applications reported by Yu Liu *et al.* [2], Erkan Beşdok [3], Guillermo Zatorre *et al.* [4], Amir Jabbari *et al.* [5], Mohamed Lamine Hafiane *et al.* [6], Kai-Wei Chiang *et al.* [7], Raúl Vicen-Bueno *et al.* [8], Juan L. Pedreño-Molina *et al.* [9], and P. B. Garcia-Allende *et al.* [10] are far more than toy applications. The lesson to be learned here is that it is a bad idea to publically bet against technological progress in computer applications.

As one paper after another arrived for my decision to accept for publishing, I was wondering if there would be any papers addressing medical applications and breast cancer in particular since that is my main research field of interest. If anyone is wondering how a medical researcher can be a guest editor of a journal dedicated to ANN, I am a classically trained experimental physicist but made a career change into cancer research a number of years ago. This was recently documented in a sister MDPI journal [11].

I know of several applications where neural networks are used in breast cancer. Peter Ravdin, MD, PhD of University of Texas developed a website that offers a free prediction of survival probability for individual patients. [12] This was based on neural networks and is meant for use by professionals. It had been trained with accurate data and has been independently tested to produce results within 2% error. This website is very frequently used by breast cancer clinicians. I also know that neural networks are used in determining whether there are any suspicious areas of concern in digital mammography images.

This is probably not going to be received favorably, but I happen to think early detection of breast cancer is not nearly as valuable as we are led to believe. Overall I think the benefits of early detection of cancer are exaggerated while the possible harms are often ignored or at least minimized. On the surface, determining the benefit of early detection would seem to be a simple computation but I can tell you that it is very complex [13].

I happen to think the mechanics of early detection of cancer is currently far more advanced than the physicians' ability to take advantage of earlier detected cancer. There needs to be improvement in treatment rather than just learning how to detect cancers earlier.

I bring this sensitive topic up since it leads me to discuss an important unsolved problem in clinical breast cancer that needs help and perhaps ANNs are the key. As it turns out, according to reports from the very competent Danish Cochrane group, 2,000 women need to be screened for cancer for 10 years in order to save one life and, as an unfortunate consequence, 10 women will be over-diagnosed and become breast cancer patients when in fact they never would have [14].

Based on the Cochrane report, it is certainly not clear whether breast cancer screening does more good than harm. All breast cancers start as malignant cells that start to divide while in the milk ducts of the breast. In that state, these are called ductal carcinoma *in-situ* or DCIS. Many of these growths eventually invade the breast tissue and can become deadly if ignored, but other DCIS never would invade the breast tissue in the host's lifetime. The number is disputed, but could be of the order of 20%–50% that never would invade. The problem stems from the fact that we currently cannot tell which DCIS need to be removed and which can safely be ignored or just watched. Approximately 20% of all mammography findings are DCIS, so it is not a small problem. Since physicians don't know which can be just watched, virtually all are treated with surgery and occasionally even further treatment is used.

Early detection is based on two assumptions. First, that tumor growth is steady and continuous and second, therapy is always more effective if applied earlier rather than later in the disease progression. As it turns out, neither assumption is always correct [15]. Breast cancer detection and treatment is a very large business and some organizations who actively promote early detection derive financial benefits from its use. Of course, there are many organizations that promote early detection and are doing it in the best altruistic interests.

Since the Cochrane report tells us that survival benefits are of the order of 0.1% of the screened population, we must worry about harmful side effects at the 0.1% level. I can tell you that side effect data at that level are not firm. This could be an excellent opportunity for development using ANN. It is not far conceptually from finding targets among sea clutter or distinguishing cloud types from satellite images [2,8]

I write this hoping to stimulate some ANN experts to tackle the problems of first, how to tell if a particular DCIS has the ability to invade or if it can just be monitored and second, how to reliably determine benefits and harms at the 0.1% level.

I want to conclude with a few general comments comparing the quality and style of papers in the engineering field and in the medical field. These are gross generalizations but nonetheless my impressions are strong. The physicians are much better communicators than are the engineers and physicists. (No doubt certain personality traits lead one towards career choices). Each sentence in a medical paper is well crafted and logically follows the preceding one and leads into the next sentence. Sentences are unambiguous. The reader is not left wondering what the writer meant.

Balancing that strength, the physicians are very poor with numerical analysis. They rely upon statisticians to tell them if their data are outside the important but arbitrary “p = 0.05” level of statistical significance. It is common in a medical journal to see data reported followed by p = 0.049. I strongly suspect that very few doctors understand the true meaning of statistical significance, but they use that all the time. I also strongly suspect that data are routinely manipulated in an attempt to report statistically significant results. There is little benefit to reporting data that are just a little outside significant levels. I strongly suspect that there is widespread abuse of statistical significance. Reporting data with p = 0.051 does not help get a grant funded and does not result in a clinical impact. At conferences, you can watch their faces as they report just barely significant data. They actually seem proud of their ability to manipulate data by discarding unwanted data or repeating data until they achieve significance. Physicians do not understand or appreciate the important distinction between random and non-random errors. Engineers on the other hand are much better at understanding and presenting data than are physicians. Engineers will typically report data in a graph showing numerical values including error bars. The reader is able to tell if intelligently presented results are significantly dominant over random noise. Physicians frequently present data in Kaplan-Meier life table curves and accompany that with data in tabular form also listing p-values. Physicians will also explain a complex biological process with cartoon drawings that rarely contain any numbers.

As a very disturbing example of physicians' inability to understand numerical data, I once saw a drug dosage reported in a paper in a major cancer journal as 0.285714 mg/week. I could not believe that they could possibly know a volume of medicine to six-figure accuracy. The number looked familiar to me. I eventually figured out that it was a repeating decimal for 2/7. The doctor was apparently transforming a 2 mg pill given once a week into a weekly dose by dividing by 7. Since his calculator gave him six decimal places, that is what he or she wrote down and none of the authors, reviewers, or editors picked it up.

I strongly suggest that engineers need to learn how to write better and doctors need to learn how to use numerical data and learn the real meaning of statistical significance.

## References

[1] Dewdney A.K. (1997). Yes, We Have No Neutrons: An Eye-Opening Tour through the Twists and Turns of Bad Science.

[2] Liu Y., Xia J., Shi C.X., Hong. Y. (2009). An Improved Cloud Classification Algorithm for China's FY-2C Multi-Channel Images Using Artificial Neural Network. Sensors.

[3] Beşdok E. (2009). 3D Vision by Using Calibration Pattern with Inertial Sensor and RBF Neural Networks. Sensors.

[4] Zatorre G., Medrano N., Sanz M.T., Aldea C., Calvo B., Celma S. (2009). Digitally Programmable Analogue Circuits for Sensor Conditioning Systems. Sensors.

[5] Jabbari A., Jedermann R., Muthuraman R., Lang W. (2009). Application of Neurocomputing for Data Approximation and Classification in Wireless Sensor Networks. Sensors.

[6] Hafiane M.L., Dibi Z., Manck O. (2009). On the Capability of Artificial Neural Networks to Compensate Nonlinearities in Wavelength Sensing. Sensors.

[7] Chiang K.W., Chang H.W., Li C.Y., Huang Y.W. (2009). An Artificial Neural Network Embedded Position and Orientation Determination Algorithm for Low Cost MEMS INS/GPS Integrated Sensors. Sensors.

[8] Vicen-Bueno R., Carrasco-Álvarez R., Rosa-Zurera M., Nieto-Borge J.C. (2009). Sea Clutter Reduction and Target Enhancement by Neural Networks in a Marine Radar System. Sensors.

[9] Pedreño-Molina J.L., Monzó-Cabrera J., Lozano-Guerrero A., Toledo-Moreo A. (2008). Design and Validation of a Ten-Port Waveguide Reflectometer Sensor: Application to Efficiency Measurement and Optimization of Microwave-Heating Ovens. Sensors.

[10] Garcia-Allende P.B., Mirapeix J., Conde O.M., Cobo A., Lopez- Higuera J.M. (2008). Arc-Welding Spectroscopic Monitoring based on Feature Selection and Neural Networks. Sensors.

[11] Retsky M. (2009). New Concepts in Breast Cancer Emerge from Analyzing Clinical Data Using Numerical Algorithms. Int. J. Environ. Res. Public Health.

[12] Olivotto I.A., Bajdik C.D., Ravdin P.M., Speers C.H., Coldman A.J., Norris B.D., Davis G.J., Chia S.K., Gelmon K.A. (2005). Population-Based Validation of the Prognostic Model ADJUVANT! for early breast cancer. J. Clin. Oncol..

[13] Keen J.D., Keen J.E. (2009). What Is the Point: Will Screening Mammography Save My Life?. BMC Med Inform Decis Mak..

[14] Gøtzsche P.C., Hartling O.J., Nielsen M., Brodersen J., Jørgensen K.J. (2009). Breast Screening: The facts—or maybe not. BMJ.

[15] Retsky M.W., Demicheli R., Hrushesky W.J., Baum M., Gukas I.D. (2008). Dormancy and Surgery-Driven Escape from Dormancy Help Explain Some Clinical Features of Breast Cancer. APMIS.

